# Clinical Outcomes in Patients With Benign Paroxysmal Positional Vertigo and Vitamin D Deficiency: A Singaporean Perspective

**DOI:** 10.7759/cureus.60325

**Published:** 2024-05-15

**Authors:** Clarisse Chu, Yew Meng Chan, Joyce Tang

**Affiliations:** 1 Otolaryngology, Singapore General Hospital, Singapore, SGP

**Keywords:** benign paroxysmal positional vertigo, giddiness, quality of life, singapore, vitamin d

## Abstract

Introduction: Benign paroxysmal positional vertigo (BPPV) is the primary vestibular disorder causing peripheral vertigo. Given the role of vitamin D in maintaining otoconia homeostasis, its deficiency may elevate the risk of BPPV. Our study seeks to evaluate the correlation between vitamin D deficiency and clinical outcomes of patients with BPPV in the local Asian population.

Methodology: We performed a retrospective analysis of 149 consecutive adult patients referred to a tertiary center's Otolaryngology dizziness clinic between 2018 and 2021. All of these patients had both BPPV and vitamin D deficiency.

Results: The mean serum vitamin D level was 19.4 ± 5.5 ng/mL. Approximately 51.7% (77/149) of patients experienced recurrent episodes of BPPV. Univariate binomial logistic regression demonstrated vitamin D levels were related to BPPV recurrence (P < 0.001); univariate chi-square analysis showed history of migraine was not significantly related to BPPV recurrence (P = 0.051). On multivariate analyses, patients with higher serum vitamin D levels had 16.7% lower odds of having a history of recurrent BPPV (odds ratio [OR] 0.83, 95% confidence interval [CI] 0.76-0.90, *P *< 0.001). However, migraine history was not significantly related to BPPV recurrence (OR 2.66, 95% CI 1.00-7.11, P = 0.0503). There was no statistically significant difference in the duration of BPPV episodes based on vitamin D levels (*P* = 0.327).

Conclusions: Among patients with vitamin D deficiency, lower serum vitamin D levels were associated with a higher odds of having a history of recurrent BPPV. Future research directions that would be beneficial include conducting a randomized controlled trial to evaluate both the effectiveness of vitamin D supplementation and its optimal dosage.

## Introduction

Benign paroxysmal positional vertigo (BPPV) affects up to 0.6% of the general population every year [1]. It is the primary vestibular disorder causing peripheral vertigo, comprising up to 20% to 30% of cases [2,3]. Individuals with BPPV typically report episodic positional vertigo, which lasts for a few seconds to several minutes. On positioning maneuvers, nystagmus is observed in the plane corresponding to the affected canal. The pathophysiology of BPPV involves canalolithiasis or cupulolithiasis, wherein displaced otoconia migrate from the macula to the semicircular canals [4,5].

In 80% of patients, BPPV is idiopathic. Risk factors in the remaining 20% include head trauma, vestibular neuronitis, and otologic surgery [6]. Recent literature indicates a potential link between vitamin D deficiency and an elevated risk of developing BPPV; however, this is yet to be evaluated in the local Asian population [7].

Otoconia contain calcium carbonate on an organic collagen matrix (otolin). It is postulated that vitamin D is a key regulator of epithelial calcium channels and calcium-binding proteins, helping to maintain endolymphatic calcium levels. As a result of vitamin D deficiency, calcium metabolism is disrupted, leading to the development of abnormal otoconia. This, in turn, contributes to the onset of BPPV [8,9].

Vitamin D deficiency is especially prevalent in Singapore, despite its equatorial location [10]. Cross-sectional studies have reported vitamin D deficiency rates of up to 42% [11]. The consequences of this deficiency include osteoporosis, muscle pain, and weakness.

Our retrospective cohort study seeks to evaluate the correlation between vitamin D deficiency and clinical outcomes of patients with BPPV in the local Asian population.

## Materials and methods

Study design and participants

This retrospective cohort study was carried out at a tertiary referral center. Approval from the SingHealth Institutional Review Board (IRB) was obtained for waiver of informed consent (IRB reference number 2021/2376). All data retrospectively collected from the medical records were de-identified to ensure anonymity.

Inclusion criteria

We identified 149 consecutive adult patients (21 years old and above), who were referred to Singapore General Hospital’s Otolaryngology dizziness clinic, between 2018 and 2021. All of the patients in this retrospective cohort study had both BPPV and vitamin D deficiency.

On initial presentation to the dizziness clinic, patients were diagnosed with BPPV if they had a classical history (positional vertigo related to head movements, lasting seconds to minutes), positive clinical examination findings (positional nystagmus with Dix-Hallpike or supine roll maneuvers, with latency and fatiguability), or both.

A history of previous BPPV was determined based on clinical documentation review, if the patient had a prior documented episode of BPPV diagnosed by an otolaryngologist, or a classical history with complete resolution of symptoms in between the previous episode and the current one for which they presented.

The diagnosis of migraine was established through a clinical documentation review, which involved confirming that the patient had either a documented medical history of migraine or, in cases where they had not previously sought treatment, had reported symptoms consistent with the diagnostic criteria for migraine with or without aura as outlined by the International Headache Society. These criteria include experiencing at least five headaches along with at least two of the following features: unilateral pain, pulsating quality, at least moderate intensity, exacerbation with routine activities, with either nausea/vomiting and/or photophobia and phonophobia.

Serum vitamin D levels were measured at initial clinical presentation, and levels below 30 ng/mL were considered deficient.

Exclusion criteria

Patients with a history of head or ear trauma, surgery, or a history of active inner ear disease were excluded. Those who had incomplete data were excluded as well.

Statistical analysis

Variables were summarized using descriptive statistics. Tests of normality for each scale variable were performed using the Shapiro-Wilk test. Categorical variables were compared using Pearson chi-square analysis, with unadjusted odds ratios derived from the corresponding contingency tables. The relationship between serum vitamin D and BPPV recurrence was assessed using univariate binomial logistic regression. The effect of various factors on BPPV recurrence was evaluated using binomial logistic regression through odds ratios (ORs) and 95% confidence interval (CI). The suitability of the model was assessed using the Hosmer-Lemeshow goodness-of-fit test. To determine statistical significance, an alpha level of 0.05 was established. IBM SPSS Statistics for Windows, Version 25.0 (IBM Corp., Armonk, NY) was used for analyses.

## Results

Patient characteristics

**Figure 1:**
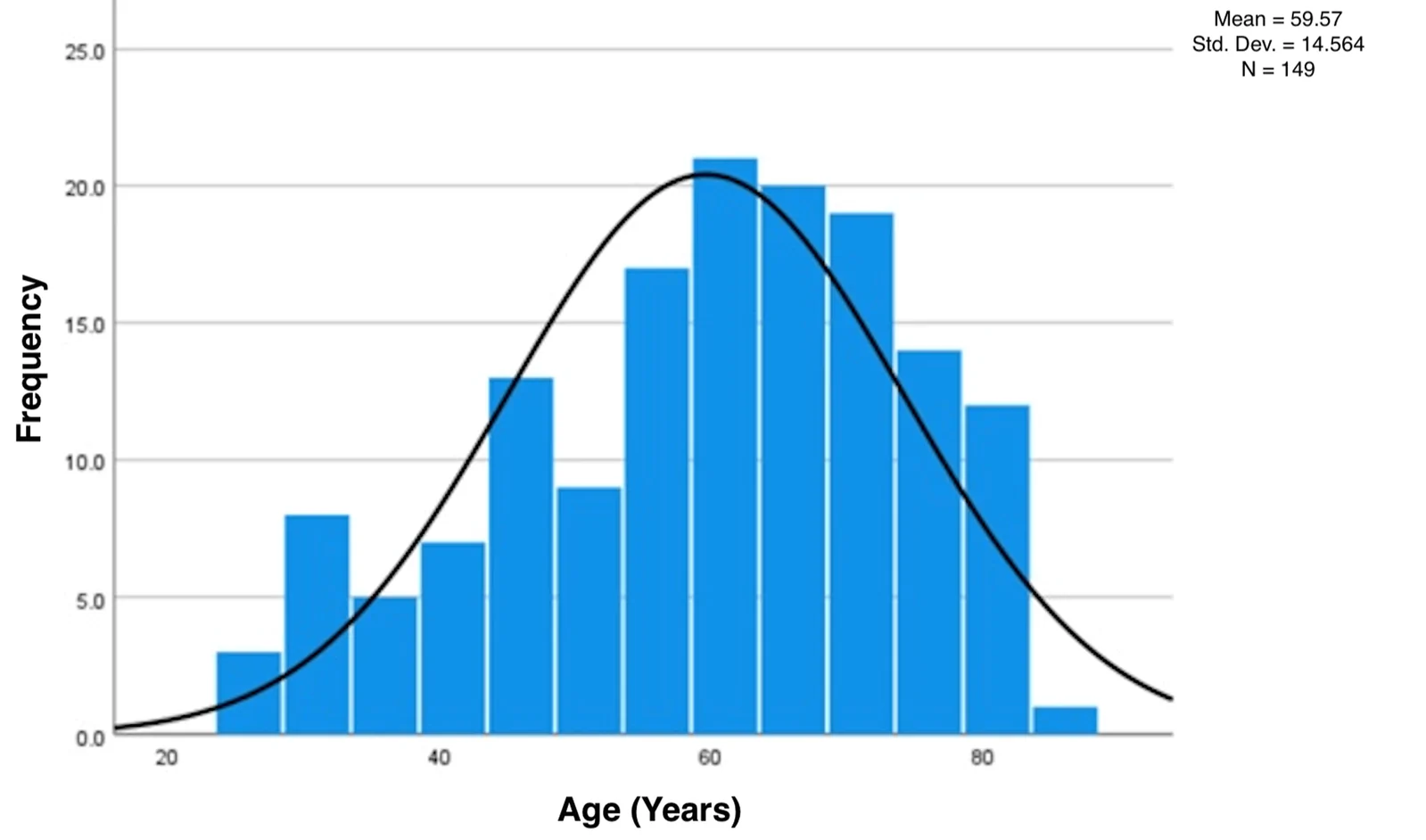
Frequency distribution of patients' ages.

An overview of the clinical and demographic features of our study cohort is given in Table 1. The largest proportion of patients were of Chinese ethnicity (118/149, 79.2%), and most were female (117/149, 78.5%). The median age was 62 (26-84) years. It exhibited a non-normal distribution (Shapiro-Wilk *P *< 0.001) (Figure 1).

**Table 1:** Patient characteristics. BPPV, benign paroxysmal positional vertigo; SD, standard deviation

Patient characteristics, *N *= 149	Values
Gender, *n *(%)	
Female	117 (78.5)
Male	32 (21.5)
Age (years), median (range)	62 (26-84)
Ethnicity, *n *(%)	
Chinese	118 (79.2)
Malay	8 (5.4)
Indian	17 (11.4)
Others	6 (4.0)
Comorbidities, *n *(%)	
Hypertension	44 (29.5)
Hyperlipidemia	47 (31.5)
Diabetes	20 (13.4)
Obstructive sleep apnea	8 (5.4)
Migraine	33 (22.1)
Vestibular conditions, *n *(%)	
Recurrent BPPV	77 (51.7)
Vestibular neuronitis	13 (8.7)
Labyrinthitis	1 (0.7)
Serum vitamin D, mean ± SD (ng/mL)	19.4 ± 5.5

**Figure 2:**
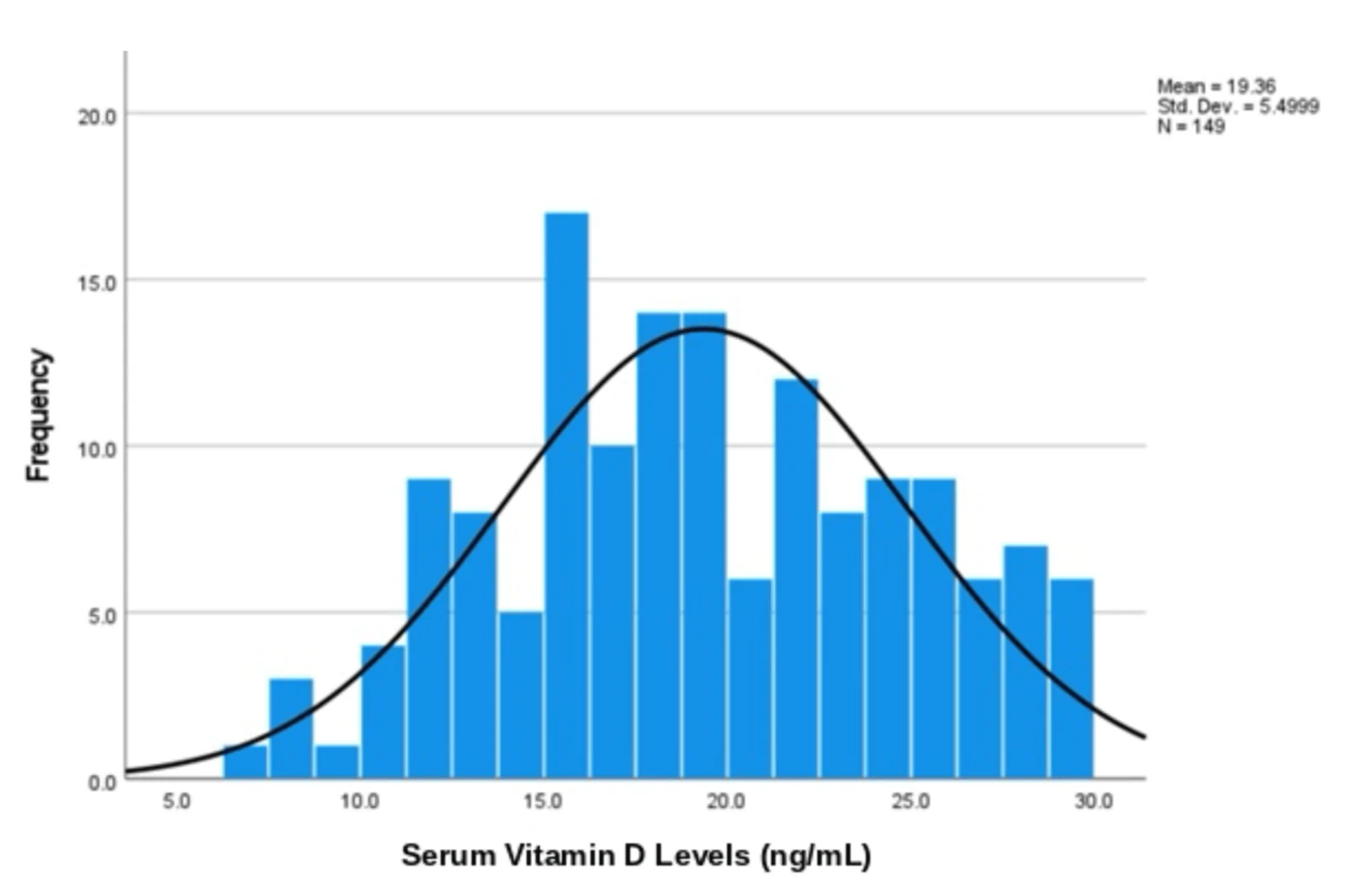
Frequency distribution of patients' serum vitamin D levels.

The mean serum vitamin D level was 19.4 ± 5.5 ng/mL; it displayed a normal distribution (Shapiro-Wilk *P *= 0.07) (Figure 2). Approximately 29.5% (44/149) had hypertension, 31.5% (47/149) had hyperlipidemia, and 13.4% (20/149) had diabetes mellitus.

Of the 149 patients, 77 (51.7%) had recurrent BPPV episodes, 13 (8.7%) had previous vestibular neuronitis, and 1 (0.7%) had previous labyrinthitis. Of the 149 patients, 33 (22.1%) had a history of migraine.

Outcome measures

**Figure 3:**
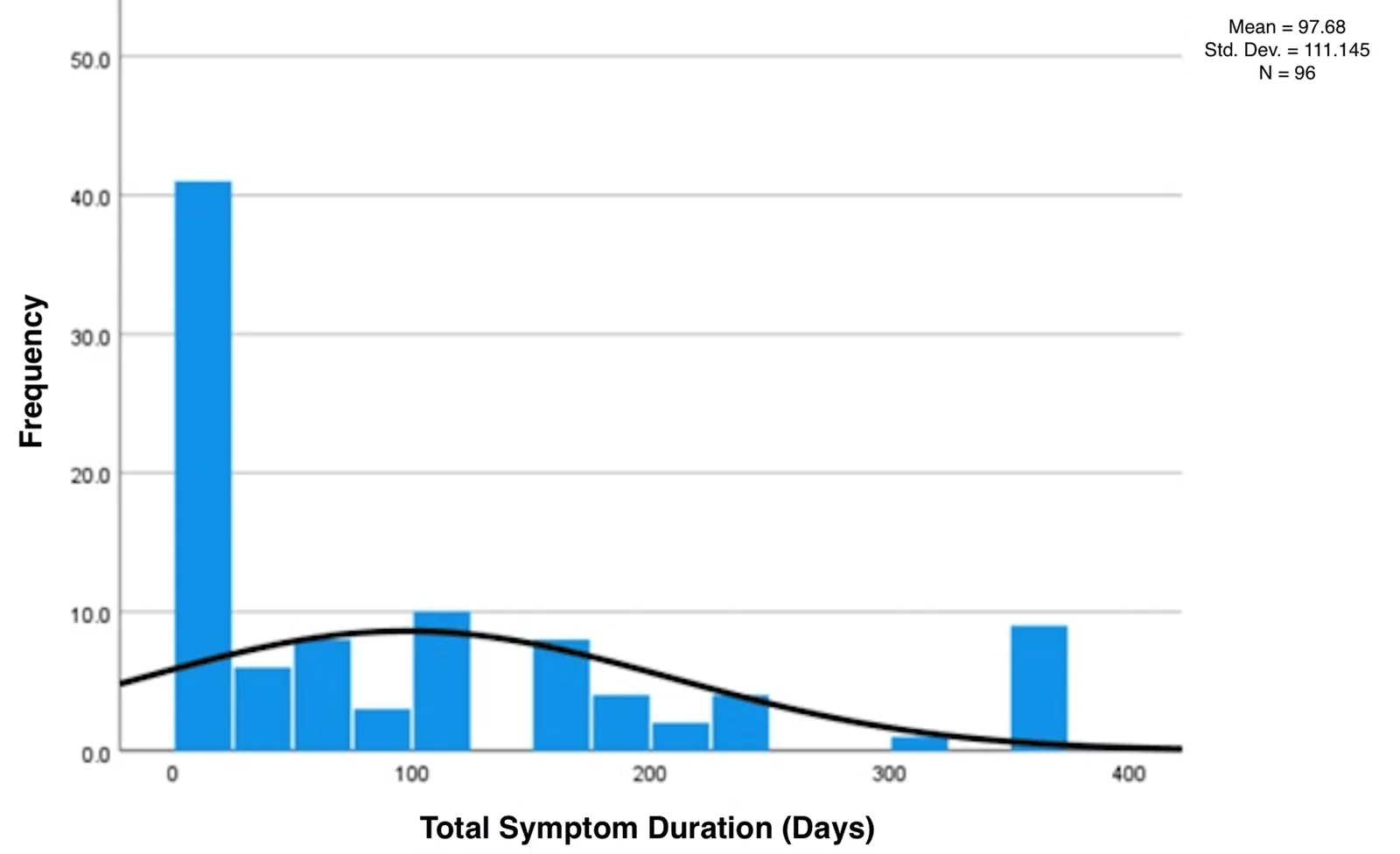
Frequency distribution of patients' total symptom duration.

The median symptom duration was 60 (1-360) days. It exhibited a non-normal distribution (Shapiro-Wilk *P *< 0.000) (Figure 3). There was an equal incidence of right- and left-sided BPPV (right 43/149, 28.9%, vs. left 42/149, 28.2%). Of the patients who had a documented canal affected (76/149, 51%), the posterior canal was involved in 68.4% (52/76), the lateral canal was involved in 22.4% (17/76), the anterior canal in 6.6% (5/76), and multiple canals in 2.6% (2/76).

Factors affecting BPPV recurrence

The factors affecting BPPV recurrence are shown in Table 2. The incidence of BPPV recurrence was 59% (23/39) in those under the age of 50, 50% (36/72) for those aged between 50 and 70 years, and 47.4% (18/38) in those aged above 70 years. Univariate chi-square analyses showed no significant association between age and BPPV recurrence, with each group compared with all other groups: for those <50 years old (OR 1.49, 95% CI 0.71-3.12, *P *= 0.29); for those 50-70 years old (OR 0.88, 95% CI 0.46-1.67, *P *= 0.69); and for those >70 years old (OR 0.79, 95% CI 0.38-1.66, *P *= 0.54).

**Table 2:** Factors affecting benign paroxysmal positional vertigo recurrence. SD, standard deviation; CI, confidence interval; BPPV, benign paroxysmal positional vertigo

Characteristics	Previous BPPV (*n* = 77)	No previous BPPV (*n* = 72)	Unadjusted		Adjusted	
			Odds ratio (95% CI)	*P*-value	Odds ratio (95% CI)	*P*-value
Gender, *n* (%)						
Female	62 (80.5)	55 (76.4)	1 (ref)	-	1 (ref)	-
Male	15 (19.5)	17 (23.6)	0.78 (0.36-1.71)	0.54	0.71 (0.28-1.79)	0.47
Age (years), median (range)						
<50 years old	23 (29.9)	16 (22.2)	1 (ref)	-	1 (ref)	-
50-70 years old	36 (46.8)	36 (50.0)	0.70 (0.32-1.53)	0.37	1.34 (0.48-3.71)	0.58
>70 years old	18 (23.4)	20 (27.8)	0.63 (0.25-1.54)	0.31	2.35 (0.65-8.50)	0.19
Ethnicity, *n* (%)						
Chinese	59 (76.6)	59 (81.9)	1 (ref)	-	1 (ref)	-
Malay	3 (3.9)	5 (6.9)	0.60 (0.14-2.63)	0.50	0.26 (0.04-1.45)	0.12
Indian	11 (14.3)	6 (8.3)	1.83 (0.64-5.28)	0.26	0.76 (0.22-2.73)	0.68
Others	4 (5.2)	2 (2.8)	2.00 (0.35-11.34)	0.43	0.63 (0.09-4.67)	0.65
Comorbidities, *n *(%)						
Hypertension	20 (26.0)	24 (33.3)	0.70 (0.35-1.42)	0.33	0.70 (0.24-2.06)	0.51
Hyperlipidemia	20 (26.0)	27 (37.5)	0.58 (0.29-1.18)	0.13	0.56 (0.19-1.58)	0.27
Diabetes	11 (14.3)	9 (12.5)	1.16 (0.45-3.00)	0.75	2.81 (0.85-9.38)	0.09
Obstructive sleep apnea	3 (3.9)	5 (6.9)	0.54 (0.13-2.36)	0.41	0.39 (0.07-2.24)	0.29
Migraine	22 (28.6)	11 (15.3)	2.21 (0.98-4.98)	0.051	2.66 (1.00-7.11)	0.050
Vestibular conditions, n (%)						
Vestibular neuronitis	8 (10.4)	5 (6.9)	1.55 (0.48-4.99)	0.46	1.39 (0.38-5.10)	0.61
Serum vitamin D (mean ± SD) (ng/mL)	17.4 ± 5.25	21.4 ± 5.02	0.86 (0.81-0.92)	<0.001	0.83 (0.76-0.90)	<0.001

The incidence of BPPV recurrence was 50% (59/118) in Chinese, 37.5% (3/8) in Malays, 64.7% (11/17) in Indians, and 66.7% (4/6) in other races. There was no significant association between ethnicity and BPPV recurrence, with each group compared with all other groups: Chinese (OR 0.72, 95% CI 0.33-1.61, *P *= 0.43); Malays (OR 0.54, 95% CI 0.13-2.36, *P *= 0.42); Indians (OR 1.83, 95% CI 0.64-5.24, *P *= 0.26); and others (OR 1.91, 95% CI 0.34-10.8, *P *= 0.46).

Gender (female, OR 1.27, 95% CI 0.58-2.80, *P* = 0.54; male, OR 0.78, 95% CI 0.36-1.71, *P* = 0.54), hypertension (OR 0.70, 95% CI 0.35-1.42, *P* = 0.33), hyperlipidemia (OR 0.59, 95% CI 0.29-1.18, *P* = 0.13), diabetes mellitus (OR 1.17, 95% CI 0.45-3.01, *P* = 0.75), and sleep apnea (OR 0.54, 95% CI 0.13-2.36, *P* = 0.41) were not significantly associated with BPPV recurrence as well, with each group compared with all other groups.

On univariate binomial logistic regression, serum vitamin D levels were related to BPPV recurrence (OR 0.86, 95% CI 0.81-0.92, P < 0.001). On univariate chi-square analysis, history of migraine (OR 2.21, 95% CI 0.98-4.98, P = 0.051) was not significantly related to BPPV recurrence.

Subsequent multivariate binomial logistic regression showed patients with higher serum vitamin D levels have 16.7% lower odds of having a history of recurrent BPPV (OR 0.83, 95% CI 0.76-0.90, *P* < 0.001). However, this analysis showed no significant association between migraine and BPPV recurrence (OR 2.66, 95% CI 1.00-7.11, P = 0.0503).

Effect of vitamin D deficiency on the duration of BPPV episodes

The characteristics of BPPV episodes are shown in Table 3. A Kruskall Wallis test showed no significant association between the duration of BPPV episodes and serum vitamin D levels (*H *= 6.88, *P* = 0.327).

**Table 3:** Characteristics of each benign paroxysmal positional vertigo episode.

Characteristics of BPPV episode, *N *= 149	Values
Laterality, *n *(%)	
Right	43 (28.9)
Left	42 (28.2)
Unknown	64 (43.0)
Canal affected, *n *(%)	
Posterior only	52 (34.9)
Lateral only	17 (11.4)
Anterior only	5 (3.4)
Multiple	2 (1.3)
Unknown	73 (49.0)
Type, *n *(%)	
Canalolithiasis	68 (45.6)
Cupulolithiasis	5 (3.4)
Symptom duration, median (range)	60 (1-360)

Factors affecting serum vitamin D levels

The factors affecting serum vitamin D levels are presented in Table 4. When stratified by age, median serum vitamin D levels were 15.9 (7.8-29.7) ng/mL in patients less than 50 years old, 19.1 (7.4-29.5) ng/mL in patients 50-70 years old, and 21.6 (13.1-29.7) ng/mL in patients above 70 years old. The incidence of BPPV recurrence was 59% (23/39) in patients aged less than 50, 50% (36/72) in those 50-70 years old, and 47.4% (18/38) in those above 70 years old. On linear regression analysis, increasing age was linked to higher levels of vitamin D, with a coefficient of *B *= 3.21 (95% CI 0.44-5.98, *P *= 0.02).

**Table 4:** Factors affecting serum vitamin D levels.

Characteristics	*B* value	95% CI	*P*-value
Gender			
Female	1 (ref)		
Male	-1.10	-3.15 to 0.94	0.29
Age			
<50 years old	1 (ref)		
50-70 years old	1.76	-0.47 to 3.98	0.12
>70 years old	3.21	0.44 to 5.98	0.02
Ethnicity			
Chinese	1 (ref)		
Malay	-2.12	-5.76 to 1.51	0.25
Indian	-5.81	-8.38 to -3.24	<0.001
Others	-4.73	-9.14 to -0.34	0.035
Comorbidities			
Hypertension	-0.72	-3.08 to 1.65	0.55
Hyperlipidemia	0.78	-1.53 to 3.08	0.51
Diabetes	1.01	-1.61 to 3.64	0.45
Obstructive sleep apnea	-2.08	-5.78 to 1.61	0.27
Migraine	-0.85	-2.95 to 1.25	0.43
Vestibular conditions			
Vestibular neuronitis	-1.90	-4.80 to 1.00	0.20

When stratified by ethnicity, median serum vitamin D levels were 20.4 (9.5-29.7) ng/mL in Chinese, 16.1 (13.4-28.1) ng/mL in Malays, 15.0 (7.4-22.7) ng/mL in Indians, and 13.9 (8.0-18.2) ng/mL in other races. Linear regression showed vitamin D levels were lower in Indians at *B* = -5.81 (95% CI -8.38 to -3.24, *P* < 0.001).

## Discussion

Our study demonstrated that, among patients with vitamin D deficiency, lower serum vitamin D levels were associated with a history of BPPV recurrence. There was, however, no direct association between serum vitamin D levels (0-30 ng/mL) and duration of BPPV exacerbations.

In countries that experience seasonal variations, vitamin D deficiency is prevalent, especially in winter months [12]. Surprisingly, even in tropical Singapore, there is a significant prevalence of vitamin D deficiency. This may be related to working long hours in indoor environments. Even among those whose offices have windows, the glass filters out UVB. As such, exposure to indoor sunlight does not result in vitamin D production [13]. Moreover, a fair complexion tends to be viewed as desirable in East Asian societies [14]. There is thus an increased propensity to engage in sun avoidance measures, such as hat-wearing, utilization of long-sleeved clothing, and application of sunscreen [15].

Our study identified a correlation between lower vitamin D levels and younger age. Possible underlying factors include increased time spent indoors during daytime hours, leading to less exposure to sunlight and UVB absorption [13]. Older adults on follow-up with primary care services for comorbidities such as osteoporosis are started on vitamin D supplementation [16]. Generally, the prevalence of BPPV rises as individuals age. However, in younger individuals, the occurrence of recurrent BPPV is uncommon, and low vitamin D levels might be a contributory factor.

Given the association observed here between lower serum vitamin D levels and recurrent BPPV, whether raising vitamin D levels reduces recurrence risk warrants prospective evaluation. Vitamin D can be derived from environmental sources such as sunlight exposure; however, it is fraught with challenges. Prolonged exposure may result in DNA damage and cutaneous malignancies; thus, exposure should be limited to low levels [17]. Equatorial nations like Singapore are subjected to intense sunlight at midday. While less intense, the morning sun contains UVA, which can cause skin reddening without vitamin D synthesis. In the context of Southeast Asia, in order to sustain adequate vitamin D levels, Nimitphong and Holick suggested a regimen of exposing the face and both arms to sunlight for 25 minutes, three times a week, starting at 9 AM [15]. Additionally, for the vitamin D deficient, there is potential for vitamin D supplementation to augment dietary sources. Future research directions that would be beneficial include conducting a randomized controlled trial to evaluate both the effectiveness of vitamin D supplementation and its optimal dosage.

The role of migraine as a risk factor for recurrent BPPV is uncertain. Zhu et al. demonstrated patients with migraine had an increased risk of developing recurrent BPPV; however, Hilton et al. showed there was no relationship [18,19]. It is postulated that migraine may result in labyrinthine artery vasospam and ischemia, causing oxidative stress and otoconia detachment from otolith organs [20]. Our study showed no significant association; however, the proportion of patients in our study population with migraine was low (*n *= 33, 22.1%). Further studies would need to be carried out to assess this relationship in our local Singaporean context.

A meta-analysis by Chen et al. in 2021 suggested that in addition to vitamin D deficiency, patients with hypertension, hyperlipidemia, and diabetes mellitus were more likely to develop recurrent BPPV [21]. However, our study did not demonstrate such a relationship. Of note, there was significant heterogeneity among the included studies. It would be beneficial to assess this correlation in larger scale cohorts.

It is interesting to note that the incidence of anterior canal BPPV in our study was 6.6%, higher than the frequency in literature of 1% to 3% [22]. The incidence of lateral canal BPPV in our center was also higher at 22.4%, compared to most reports of 10% to 12% [23].

The strength of this study is uniform documentation of patient records in a single dizziness clinic. To the best of our knowledge, our study is the first to explore the relationship between vitamin D deficiency and BPPV in the local Singaporean and South East Asian context. Given the incidence of vitamin D deficiency and dizziness, this is an important springboard for further clinical trials.

Limitations of this study include retrospective data collection from our cohort. Additionally, all patients had their serum vitamin D levels assessed on first presentation to the otolaryngology dizziness clinic, but not all had a repeat serum vitamin D level tested. As such, our analysis only utilized the first vitamin D reading. Serum vitamin D was measured at presentation to the dizziness clinic, whereas previous BPPV episodes were identified retrospectively. As vitamin D levels were not available at the time of the earlier BPPV episodes, the temporal relationship between vitamin D status and recurrence could not be directly assessed.

## Conclusions

In conclusion, BPPV and vitamin D deficiency are especially prevalent in Singapore, causing economic burden and loss of quality of life. This is the first study on vitamin D deficiency and BPPV in the local Singaporean and South East Asian context. Among patients with vitamin D deficiency, lower serum vitamin D levels were associated with a higher odds of having a history of recurrent BPPV. These findings serve as a springboard for future clinical trials, which include evaluating both the effectiveness of vitamin D supplementation and its optimal dosage.
