# Correction: Clinical Outcomes in Patients With Benign Paroxysmal Positional Vertigo and Vitamin D Deficiency: A Singaporean Perspective

**DOI:** 10.7759/cureus.c473

**Published:** 2026-08-19

**Authors:** Clarisse Chu, Yew Meng Chan, Joyce Tang

**Affiliations:** 1 Otolaryngology, Singapore General Hospital, Singapore, SGP

Following post-publication correspondence from a reader, the authors and the journal have reviewed this article for errors. As part of that review, the authors re-ran the relevant analyses in full on the original study dataset, and the editorial office independently verified the resulting output against the published article. The review found that the study’s data and analyses are sound, but that a number of errors were introduced in preparing the manuscript, and that some of the article’s wording claimed more than the study design can support. The corrections described below do not change any statistical inference in the article.

**Transcription and typographical errors. **Several values were transcribed incorrectly from the statistical output. The most consequential concern was the adjusted odds ratio for migraine: the Abstract and the Results text reported values (0.38 and 0.83) that differ from the correct estimate given in Table 2, which is OR 2.66 (95% CI 1.00-7.11; P = 0.0503). The erroneous values lay on the opposite side of unity from the correct one; readers should rely on the corrected estimate, noting that this association was, and remains, statistically non-significant. In Table 2, a decimal-placement error in one confidence interval and a duplicated confidence interval in another row, which the latter identified and reported by the authors themselves, have been corrected. The unadjusted P value for migraine, which sits at the significance boundary, is now reported exactly (P = 0.051). Two further typographical errors in the Results have also been corrected. Throughout the article, the units of serum vitamin D, which appeared as “ng/mol”, have been corrected to ng/mL, consistent with the Methods and the source data, and Figure 2 has been replaced accordingly.

**Description of the statistical methods. **The univariate analyses were previously attributed to chi-square testing alone, which cannot produce the odds ratios reported. The article now describes the methods as performed: univariate binomial logistic regression for continuous serum vitamin D, and Pearson chi-square analysis for categorical variables, with unadjusted odds ratios derived from the corresponding contingency tables. A sentence stating this has been added to the Methods, and a clarification has been added where the Results text compares each group with all other participants while Table 2 uses a single reference category; both presentations are correct.

**Scope of the conclusions. **Because every participant in this study had vitamin D deficiency at enrollment, the study compares degrees of deficiency and cannot estimate the effect of deficiency relative to vitamin D-sufficient individuals. In addition, serum vitamin D was measured at presentation, whereas previous BPPV episodes were identified retrospectively, so the temporal relationship between vitamin D status and recurrence could not be assessed. The Abstract, Conclusions and Discussion have been revised to state that, among patients with vitamin D deficiency, lower serum vitamin D levels were associated with a higher odds of having a history of recurrent BPPV, and a corresponding limitation has been added.

